# Sex differences in childhood maltreatment, inflammation, and adulthood depression: A network analysis

**DOI:** 10.1016/j.bbih.2023.100611

**Published:** 2023-03-01

**Authors:** Jay D. O'Shields, Brian D. Graves, Orion P. Mowbray

**Affiliations:** University of Georgia, School of Social Work, USA

**Keywords:** Inflammation, Major depression, Childhood maltreatment, Cytokines, Endothelial markers

## Abstract

**Background:**

Efforts to improve treatment for adults with major depression (MD) and childhood maltreatment (CM) have identified inflammation as a potential target to improve health. Network models have emerged as a new way to understand the relationship between depressive symptoms and inflammation. However, none have accounted for the role of childhood maltreatment in the link between depressive symptoms and inflammation, or sex differences commonly found in these constructs.

**Methods:**

Data from two waves of the Midlife Development in the United States study were used in this study (N = 1917). The Center for Epidemiological Studies Depression (CES-D) scale and Childhood Trauma Questionnaire, and six inflammation markers served as nodes in an undirected psychometric network analysis. Edges between nodes were calculated using partial Spearman's correlation. Separate networks were modeled for males and females.

**Results:**

The total network revealed several associations between nodes of CM, MD, and inflammation, with emotional abuse having a strong association with somatic complaints. Network comparison testing revealed male-female network invariance, with several edge differences between male and female networks. Males and females showed differences in associations across inflammatory markers and depressive symptom clusters, particularly among somatic complaints and interpersonal difficulties.

**Conclusions:**

Specific associations between dimensions of inflammation, CM, and MD may represent important targets for treatment. Network models disaggregated by sex showed that males and females may have fundamentally different associations between these constructs, suggesting that future studies should consider sex-specific interventions.

## Highlights

• Major depression, childhood maltreatment, and inflammation are complex systems.
• Network analyses showed that males and females have differences in these systems.
• Emotional abuse is associated with somatic complaints across sexes.
• Sex differences emerged across several inflammation markers and depressive symptoms.

## 1 Introduction

Major depression (MD) is a common mental health problem and a leading cause of disability, affecting 10.4% of US adults annually (Hasin et al., 2018). Additionally, episodes of MD are typically moderate to severe, recurrent, and frequently associated with medical comorbidities like coronary artery disease and diabetes mellitus (Gold et al., 2020; Otte et al., 2016). Further, MD is difficult to treat, with under 50% of individuals affected receiving effective treatment and even fewer experiencing sustained remission (Machado et al., 2006; Pigott, 2015). Regrettably, outcomes are significantly worse for those with a history of childhood maltreatment (CM), and moderate to severe CM history is present in an estimated 57.1% of individuals who experience MD in adulthood (Gardner et al., 2019; Teicher et al., 2022). A history of CM is associated with earlier onset of MD, greater symptom severity, poorer response to treatment, an increased risk for suicide, and a more chronic course (Angelakis et al., 2019; Harkness et al., 2012; Nelson et al., 2017). Thus, understanding how CM affects adults who experience MD may hold significant promise for identifying novel treatment targets.

A common link between CM and MD in adulthood is a dysregulation of inflammation. Recalled CM has been associated with both individual inflammation markers such as C-reactive protein (CRP), fibrinogen, interleukin-6 (IL-6), and white blood cell count, as well as composites of multiple inflammation markers (Danese et al., 2007; Danese et al., 2008; O'Shields et al., 2022). Additionally, CRP as an index of inflammation has been associated with several aspects of MD such as risk for antidepressant resistance, increased risk for psychiatric hospitalization, increased risk for number of depressive symptoms, and an increased risk to experience psychosocial difficulties due to depressive symptoms (Haroon et al., 2018; Köhler-Forsberg et al., 2017; O'Shields and Mowbray, 2021; Wium-Andersen et al., 2013). Recalled CM is associated with increased CRP, fibrinogen, and white blood cell count in adulthood, independent of co-occurring early life risk factors, adulthood stress, and adult health and health behaviors (Danese et al., 2007). This connection is further supported by recent findings related to immunometabolism in which studies have identified that early life adversity such as CM may alter mitochondrial at a functional and structural level (Zitkovsky et al., 2021). These mitochondrial alterations have also been found to affect the production of inflammatory molecules in the periphery as well as increase the energy expenditure needed to activate this response in those who experience MD.

Despite this evidence, current models lack a nuanced approach to understanding how these constructs interact as part of a system. Individuals treated for decreased mood as a method of preventing the onset of MD have lower rates of MD, suggesting that decreased mood may play an etiological role in the development of other depressive symptoms (Muñoz et al., 2012). The role of IL-6 in the stimulation of the liver to release CRP and fibrinogen, as well as the role of CRP in the stimulation of tumor necrosis factor-alpha (TNF-α) have long been documented (Du Clos, 2000; Kerr et al., 2001; Papanicolaou et al., 1998). Furthermore, IL-6 and TNF-α are associated with increased intercellular adhesion molecule-1 (ICAM-1) gene expression, and soluble ICAM-1 (sICAM-1) has been found to be associated with modulation of the blood brain barrier (BBB; Müller, 2019; Wung et al., 2005). Thus, increased ICAM-1 in people experiencing MD or a history of MD may help explain cytokines crossing the BBB and inducing depressive symptoms (Müller, 2019; Slavich and Irwin, 2014). Further, CM shows similar complexity, with individuals often experiencing multiple instances of CM or experiencing multiple types of CM concurrently (Claussen and Crittenden, 1991; Hahm et al., 2010; Matsumoto et al., 2021). However, CM characterized by deprivation (such as neglect and institutionalization) are associated with changes in the brain related to social processing, whereas CM characterized by threat (such as community violence and physical abuse) are associated with changes in the brain related to heightened threat detection (McLaughlin et al., 2014). Thus, the combined experience of deprivation and threat may be associated with a more frequent and elongated stress response relative to individuals experiencing one type of CM or no CM at all (McLaughlin et al., 2014). Therefore, stress responsivity is likely greater in individuals with a history of deprivation and threat relative to those that only experience one type of CM. In summation, selecting a single marker to represent inflammation, or the collapsing of markers, depressive symptoms, and maltreatment experiences into their respective global measures belies the complexity of these interacting systems.

Further, few studies have evaluated the role of sex in the complex relationship between CM, MD, and inflammation, despite significant evidence of dimorphism. Females have consistently been found to have higher rates of MD starting at the onset of puberty (Slavich and Sacher, 2019; Hyde et al., 2008), with one potential reason for this difference being that females tend to have a greater pro-inflammatory response to immunological challenge. However, a greater inflammatory response has not been uniformly associated with greater depressive symptoms across males and females with some hypothesizing that sex-hormones play a contributing role (Epskamp et al., 2016; Moieni et al., 2015; Vogelzangs et al., 2012; Slavich and Sacher, 2019; Bekhbat and Neigh, 2018). Sex-hormones can bind to immune cells and lead to complex pro- and ant-inflammatory effects (Slavich and Sacher, 2019). Briefly, the four major types of estrogen (estrone, 17beta-estradiol, estriol, and estetrol) have been found to have a U-curved effect of action in which low concentrations are associated with pro-inflammatory functions and low concentrations are associated with anti-inflammatory functions (Slavich and Sacher, 2019). Further, some evidence suggests that estrogen may be protective against BBB disruption, potentially protecting against increased concentrations of inflammatory molecules commonly associated with CM (Sohrabji, 2007).

### 1.1 Present study

Network analysis has recently emerged as a method for analyzing high dimensional data while embracing the complexity between variables. This method analyzes how variables act as nodes in a network with conditional relationships between them being modeled as edges (Borsboom et al., 2021). Recent applications of network modeling have included potentially interacting systems such as between disorders (i.e. anxiety and MD) and between different levels (i.e. psychological and neurological; Bassett and Sporns, 2017; Contreras et al., 2019). Additionally, network models have evolved as a popular method for understanding symptom level data of specific mental health problems, with a recent review citing 58 networks for MD, alone (Malgaroli et al., 2021).

Among the strong number of network studies already published, a significant branch of research using network analysis to account for how inflammation may influence depressive symptoms has emerged (Aruldass et al., 2021; Kitzbichler et al., 2021; Fried et al., 2020; Moriarity et al., 2021; Kappelmann et al., 2021; Manfro et al., 2022; Chalmers et al., 2022). Regrettably though, studies have not accounted for the effects of CM. Furthermore, the role of sex has not been robustly investigated with work by Fried et al. (2020) noting a positive association between CRP and sex, but not IL-6 or TNF-α, and work by Moriarity et al. (2021) only including sex as a background variable along with several other relevant controls and not accounting for direct effects. Only Chalmers et al. (2022) have thus far estimated separate male and female networks, finding that none of their included inflammatory molecules (CRP, IL-6, IL-8, IL-10, IL-12) were associated with depression for males, but that IL-6 was associated with MD for females. Thus, the present study sought to answer two core questions.

1. What is the network level relationship among MD and inflammation markers when accounting for CM?
2. Is there significant dimorphism among sex-disaggregated network models?

## 2 Method

### 2.1 Sample

The present study is a secondary data analysis of the Midlife Development in the United States (MIDUS) study. First started in 1995, the MIDUS study is a multi-wave, multidisciplinary survey of adults in the U.S., aimed at understanding differences in age related health and well-being (Brim et al., 2020). The present study draws from the biomarker sub-projects of Wave 2 (collected from 2004 to 2009) and the biomarker sub-projects of the Refresher Wave (collected from 2012 to 2016; Ryff et al., 2019; Weinstein et al., 2019). The biomarker sub-project consisted of participants that completed the main survey of either wave and were agreeable to an overnight visit at either the University of Wisconsin, University of California Los-Angeles, or Georgetown University. This allowed for the collection of biological measures and additional psychological testing not accounted for by the main survey. The Wave 2 biomarker sub-project yielded a sample size of 1255 participants, whereas the Refresher wave biomarker sub-project yielded a sample size of 863. The present study pooled both waves to create a potential analytic sample size of 2118 participants.

### 2.2 Measures

*Depressive Symptoms* were measured using the Center for Epidemiological Studies Depression Scale (CES-D; Radloff, 1977). The CES-D is a 20-item measure of depressive symptoms with participants' endorsement of each item ranging from 0 (rarely or none of the time) to 3 (most or all of the time) over the past 7 days. Subscales include depressive affect (range: 0–20), positive affect (range: 0–12), somatic complaints (range: 0–18), and interpersonal difficulties range (range: 0–6). Several prior studies have utilized the CES-D for the measurement of MD in the context of both maltreatment and related inflammation (Cassidy-Bushrow et al., 2012; Kisely et al., 2018; O'Shields et al., 2022). Cronbach's alpha for the CES-D using data from the total analytic sample was 0.890. Cronbach's alpha for male and female subsets were 0.892 and 0.889, respectively.

*Recalled Childhood Maltreatment* was measured using the Childhood Trauma Questionnaire (CTQ; Bernstein et al., 2003). The CTQ is a retrospective measure of maltreatment before the age of 18 across five subtypes of maltreatment: emotional abuse, physical abuse, sexual abuse, emotional neglect, physical neglect. Each subtype is measured by five probes for an experience of maltreatment with possible responses ranging from 1 (never true) to 5 (very often true). Prior research has found that each subtype of the CTQ is significantly associated with MD in adulthood, with the strongest associations being noted between emotional abuse and MD (Humphreys et al., 2020). Additionally, a meta-analysis of 25 studies, 12 of which utilized the CTQ, showed a significant association between CM and several measures of inflammation in adulthood (Baumeister et al., 2016). Cronbach's alpha for the CTQ using data from the total analytic sample was 0.930. Chronbach's alpha for male and female subsets were 0.905 and 0.939, respectively.

*CRP, fibrinogen, IL-6, TNF-α, sICAM-1, soluble E-selectin (sE-selectin)* were included as markers of inflammation. Samples were obtained via fasting blood draw on the second day of the university visit from the hours of 0630–0700. Participants were asked to avoid any form of strenuous activity prior to the blood draw. CRP, IL-6, and TNF-α were assayed via immunoelectrochemiluminescence. sE-selectin and sICAM-1 were assayed by enzyme-linked immunosorbent assay. Fibrinogen was measured via immunoturbidimetric assay. Laboratory methods did not change between the MIDUS 2 biomarker subproject and the MIDUS Refresher biomarker subproject. Laboratory methods for included inflammatory markers have been included as a part of supplementary materials (Supplemental Table 1). All laboratory methods are publicly available through the Inter-university Consortium for Political and Social Research (Ryff et al., 2019; Weinstein et al., 2019). Each inflammation marker selected has been previously found to be associated with CM, MD, or both CM and MD (Dion-Albert et al., 2022; Müller 2019; Slavich and Irwin, 2014). Additional work has also identified that each of the included markers partially mediates the association between CM and MD when modeled as a latent construct of inflammation, and CRP, IL-6, and TNF-α have been found to be associated with MD symptoms when measured as part of a network model (Fried et al., 2020; Moriarity et al., 2021; O'Shields et al., 2022; Manfro et al., 2022; Chalmers et al., 2022).

*Age, income, body mass index (BMI), and past month alcohol consumption* were included as covariates. Age has previously been shown to have a significant negative effect on inflammation and therefore was included as continuous measure with a range from 25 to 84 years. BMI was included as a continuous measure ranging from 14.99 to 77.58. Annual household income was selected to control for socioeconomic status, which ranged from $0 to $300,000.00 USD. Any income greater than 300,00.00 USD was top-coded during MIDUS data collection to 300,000.00 to protect the identity of participants. Alcohol use was a categorical measure defined as the frequency of past month alcohol consumption. Responses ranged from 1 (no alcohol) to 6 (everyday alcohol consumption).

*Sex* was included as a self-report measure by asking participants if they identified as male or female.

### 2.3 Analysis plan

The present study consisted of two complementing phases of analyses, conducted using R version 4.1.2 (R core team, 2021). Univariate statistics were calculated to characterize the sample. To understand the relationships between depressive symptoms, recalled CM, and inflammation while controlling for covariates, an undirected weighted psychometric network model was estimated using the EBICglasso algorithm via the qgraph package (Epskamp et al., 2016). This algorithm regularizes a correlation matrix using the graphical least absolute shrinkage and selection operator (glasso) at varying levels of regularization across a number of network models. The extended Bayesian information criterion (EBIC) is then utilized to select the network model with the best fit. Notably, EBIC computation is modified by a hypertuning parameter, γ, which controls for the degree of penalization for model complexity. For the present study, we utilized a pair-wise spearman's ρ for the input correlation matrix to minimize any transformation or distortion of the data, estimated 100 networks at varying levels of glasso regularization, and set γ to 0.5. The resulting network displays associations (edges) between variables (nodes) as a set of regularized partial correlations in which the smallest edges are shrunk to zero to control for spurious associations and the remining edges can be interpreted as conditionally dependent. To assess the accuracy and reliability of the edge weight estimates, we utilized a non-parametric bootstrapping procedure using 2500 bootstraps. All variables were entered into the network model simultaneously.

Node strength, closeness, and betweenness centrality measures were calculated to understand the relationship between nodes in the model. Strength measured how influential a node is to the network by taking the absolute value of all edges for a given node and can be interpreted as how directly influential a node is to other nodes in the network (Costantini et al., 2015). Closeness measured how well a node was indirectly connected to other nodes by calculating the inverse of the sum of the shortest path between nodes and can be interpreted as nodes most likely to be influenced by changes to other nodes in the network (Costantini et al., 2015). Betweenness measured how often a given node lies on the shortest path connecting any other two nodes and can be interpreted as understanding which nodes are likely to act as bridges between other nodes in a network (Costantini et al., 2015). Reliability of centrality measures were estimated using a bootstrap procedure with 2500 bootstraps, with resulting correlation stability coefficients (CS) being preferably greater than 0.5 and minimally greater than 0.25 (Epskamp et al., 2016).

The second phase consisted of extending these methods to explore potential sex differences. The analytic sample was disaggregated by sex, before again calculating univariate statistics. Mean differences across variables between males and females were calculated using the Mann-Whitney *U* Test. The same network estimation approach was then replicated for both male and female identifying participants. Networks were then compared using the Network Comparison Test to assess if males and females have differing associations between nodes (van Borkulo et al., 2022; see Haslbeck (2022) for a simulation study of network comparison methods). This method allows for the testing of two differences between networks: 1) network invariance to test if there are significant differences between the edges of two networks and 2) network global strength to test if there are differences in overall network connectivity.

Because CRP levels greater than 10 μg/mL may be indicative of acute illness and thus not a reliable measure of inflammation related to psychosocial events, participants with levels greater than 10 μg/mL were removed from the sample (n = 134). Therefore, the final analytic sample consisted of 1984 participants, 93.67% of the potential study sample. Of the final analytic sample, 96.77% of participants provided complete data. Given the low missingness analyses were carried out treating missing data as missing at random, and pairwise correlations were utilized to retain the maximal number of participants when the network models (Schafer, 1999; Dong and Peng, 2013). Additionally, to further test the sensitivity of results, we re-tested the total sample network model with CRP levels greater than 10 μg/mL included.

## 3 Results

### 3.1 Aggregate sample description

The final sample included 920 males (46.37%) and 1064 females (53.62%) with a mean age of 53 years (SD = 12.574). A mild to moderate degree of depressive symptoms (M = 15.299, SD = 5.264) and recalled CM (M = 38.114, SD = 14.136) were present. Emotional neglect and emotional abuse scores were the most prominent forms of maltreatment with an average score of 9.761 (SD = 4.521) and 8.048 (SD = 4.095), respectively. Mean CRP levels were found to be 2.165 μg/mL with a standard deviation of 2.171 μg/mL, indicating that less than one standard deviation above the mean would be above the typical cut-off of 3 μg/mL used to indicate low-grade inflammation. Table 1 contains a full review of sample descriptive statistics.

**Table 1:** Sample descriptive statistics.

	M (SD) or n	Range or %
CES-D sum	15.299(5.264)	0.00–45.00
CES-D-DA	1.984(2.706)	0.00–20.00
CES-D-SC	3.616(3.177)	0.00–18.00
CES-D-I	0.444(0.865)	0.00–6.00
CES-D-PA	9.255(2.706)	0.00–12.00
CTQ sum	38.114(14.136)	25.00–121.00
Emotional Abuse	8.048(4.095)	5.00–25.00
Emotional Neglect	9.761(4.521)	5.00–25.00
Physical Abuse	6.981(3.034)	5.00–25.00
Physical Neglect	6.824(2.702)	5.00–24.00
Sexual Abuse	6.523(3.881)	5.00–25.00
CRP (ug/mL)	2.165(2.171)	0.020–9.980
Il-6 (pg/mL)	1.143(3.675)	0.060–145.050
TNF-α (pg/mL)	2.178(1.159)	0.310–39.190
Fibrinogen (pg/mL)	340.429(76.755)	45.000–789.000
sICAM-1 (ng/mL)	276.749(144.188)	2.810–3334.920
sE-selectin (ng/mL)	41.553(20.743)	0.090–178.050
Income	77,542.69(62,553.30)	0.00–300,000.00
Age	53.106(12.574)	25–84
BMI	29.535(6.552)	14.990–77.580
Alcohol Consumption	2.571(1.547)	1–6
Sex		
Male	920	46.37%
Female	1064	53.62%

CES-D: Center for Epidemiologic Studies Depression Scale; DA: depressed affect; SC: somatic complaints; I: interpersonal difficulties; PA: positive affect; CTQ: Childhood Trauma Questionnaire; CRP: C-reactive protein; IL-6: interleukin-6; TNF-α: tumor necrosis factor-alpha; sICAM-1: soluble intercellular adhesion molecule-1; sE-selectin: soluble E-selectin; BMI: body mass index.

### 3.2 Aggregate network model analysis

**Fig. 1:**
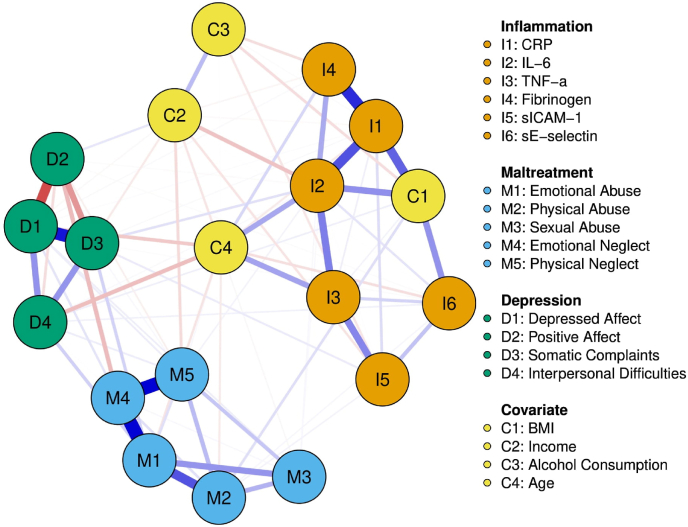
Network analysis of CM subtypes, MD symptom clusters, inflammatory molecules, and covariates Nodes represent variables and edges represent assocaitons. Edges in red denote a negative association where as edges in blue denote a positive association. (For interpretation of the references to colour in this figure legend, the reader is referred to the Web version of this article.)

Fig. 1 shows the relationship between depressive symptoms, CM, inflammation markers, and covariates for the total network model, as well as associated centrality measures. The total network model is comprised of 19 nodes with 86 non-zero edges among them and a mean weight of 0.023 among associations. Many nodes tended to cluster strongly within their own construct, most notably: depressed affect and somatic complaints (0.368), emotional abuse and emotional neglect (0.399), emotional neglect and physical neglect (0.402), and CRP and fibrinogen (0.334). Across constructs, emotional abuse was associated with somatic complaints (0.071) and interpersonal difficulties (0.075), somatic complaints were associated sICAM-1 (0.033) and IL-6 (0.047), and depressed affect was associated with fibrinogen (0.082). Among covariates, BMI showed an association with CRP (0.248), IL-6 (0.175), and sE-selectin (0.170), while age was associated with IL-6 (0.138), TNF-α (0.144), and interpersonal difficulties (−0.100). Reliability analysis indicated that node strength centrality had excellent stability (CS(cor = 0.7) = 0.750), with IL-6 (1.633), emotional abuse (1.522), depressed affect (1.114), and CRP (1.107) having the highest strength. Node centrality closeness had acceptable stability (CS(cor = 0.7) = 0.517), with IL-6 (1.539) and age (1.818) having the highest closeness. High node centrality betweenness was noted among emotional abuse (1.817), IL-6 (1.817), and age (1.734). Betweenness (CS(cor = 0.7) = 0.283) centrality had poor stability and should not be interpreted as reliable. All network edge coefficients, plotted bootstrapped edges, and centrality coefficients can be reviewed in supplemental materials (Supplemental Figs. 1 and 2, Supplemental Tables 2 and 3).

### 3.3 Sex disaggregate sample comparison

Descriptive statistics disaggregated by sex can be reviewed in Table 2. Notable differences include females reporting greater depressed affect (M = 2.231, SD = 3.264, p < 0.001), somatic complaints (M = 3.801, SD = 3.282, p < 0.05), emotional abuse (M = 8.608, SD = 4.591, p < 0.001), and sexual abuse (M = 7.363, SD = 4.826, p < 0.001). Males were found to have higher levels of sE-selectin (M = 43.720 ng/mL, SD = 21.554 ng/mL, p < 0.001) and TNF-α (M = 2.227 pg/mL, SD = 0.847 pg/mL, p < 0.001), while women were found to have higher levels of CRP (M = 2.495 μg/mL, SD = 2.365 μg/mL, p < 0.001) and fibrinogen (M = 351.276 pg/mL, SD = 78.152 pg/mL, p < 0.001).

**Table 2:** Sample descriptive statistics disaggregated by sex with mean comparisons.

	Female		Male		U	Effect Size
	M (SD)	Range	M (SD)	Range		
CES-D sum	15.747(5.381)	0.00–43.00	14.783(5.080)	0.00–45.00	434082.5***	−0.110
CESD-DA	2.231(3.264)	0.00–18.00	1.700(2.885)	0.000–20.000	431683.5***	−0.115
CESD-PA	9.284(2.703)	0.00–12.00	9.220(2.711)	0.000–12.000	478927	−0.018
CESD-SC	3.801(3.282)	0.00–18.00	3.404(3.039)	0.000–17.000	455861*	−0.065
CESD-I	0.432(0.828)	0.00–5.00	0.459(0.906)	0.000–6.000	491300	0.007
CTQ sum	39.825(16.201)	25.00–121.00	36.148(10.991)	25.00–87.00	450253**	−0.068
Emotional Abuse	8.608(4.591)	5.00–25.00	7.402(3.323)	5.00–24.00	426402.5***	−0.125
Emotional Neglect	9.904(4.784)	5.00–25.00	9.596(4.194)	5.00–25.00	484751	−0.005
Physical Abuse	7.073(3.299)	5.00–25.00	6.874(2.692)	5.00–25.00	510543	0.046
Physical Neglect	6.900(2.908)	5.00–24.00	6.737(2.440)	5.00–19.00	496128.5	0.017
Sexual Abuse	7.363(4.826)	5.00–25.00	5.557(1.969)	5.00–23.00	389001.5***	−0.195
CRP (ug/mL)	2.495(2.365)	0.050–9.980	1.785(1.852)	0.020–9.910	406236***	−0.170
Il-6 (pg/mL)	1.200(4.668)	0.170–145.050	1.076(1.981)	0.060–49.920	478737	−0.019
TNF-α (pg/mL)	2.135(1.372)	0.310–39.190	2.227(0.847)	0.510–9.520	540408.5***	0.108
Fibrinogen (pg/mL)	351.276(78.152)	45.000–789.000	327.885(73.169)	94.000–621.000	404535.5***	−0.173
sICAM-1 (ng/mL)	280.501(171.029)	2.810–3334.920	272.409(104.831)	30.000–1285.000	484175	−0.009
sE-selectin (ng/mL)	39.681(19.836)	0.090–166.970	43.720(21.554)	7.400–178.050	547549***	0.121
Income	69,503.59(58,581.79)	0.00–300,000.00	86,081.59(65,642.33)	0.00–300,000.00	542647.5***	0.164
Age	52.388(12.253)	25–84	53.936(12.892)	25–83	525255.5**	0.073
BMI	29.425(7.215)	14.990–64.060	29.662(5.690)	18.840–77.580	525536.5**	0.074
Alcohol	2.289(1.362)	1–6	2.897(1.660)	1–6	590508***	0.206

CES-D: Center for Epidemiologic Studies Depression Scale; DA: depressed affect; SC: somatic complaints; I: interpersonal difficulties; PA: positive affect; CTQ: Childhood Trauma Questionnaire; CRP: C-reactive protein; IL-6: interleukin-6; TNF-α: tumor necrosis factor-alpha; sICAM-1: soluble intercellular adhesion molecule-1; sE-selectin: soluble E-selectin; BMI: body mass index.

*P < 0.05.

**P < 0.01.

***P < 0.001.

All mean comparisons measured by Mann-Whitney test (U).

Effect sizes are reported as rank-biserial correlations.

### 3.4 Sex disaggregated network models

**Fig. 2:**
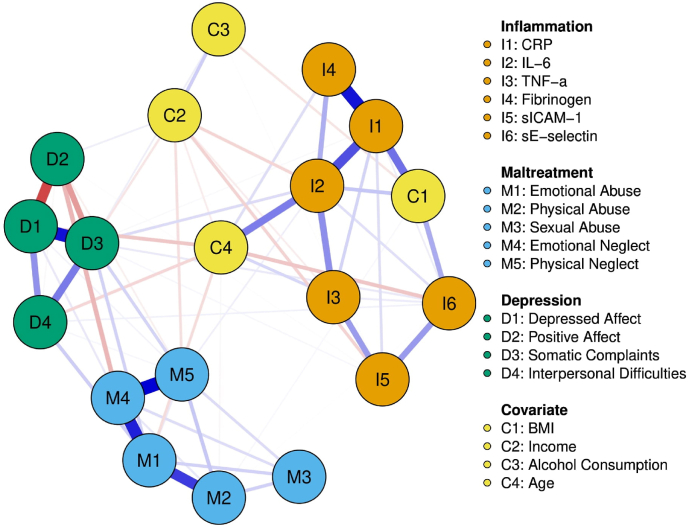
Network analysis of CM subtypes, MD symptom clusters, inflammatory molecules, and covariates for male participants Nodes represent variables and edges represent assocaitons. Edges in red denote a negative association where as edges in blue denote a positive association. (For interpretation of the references to colour in this figure legend, the reader is referred to the Web version of this article.)

**Fig. 3:**
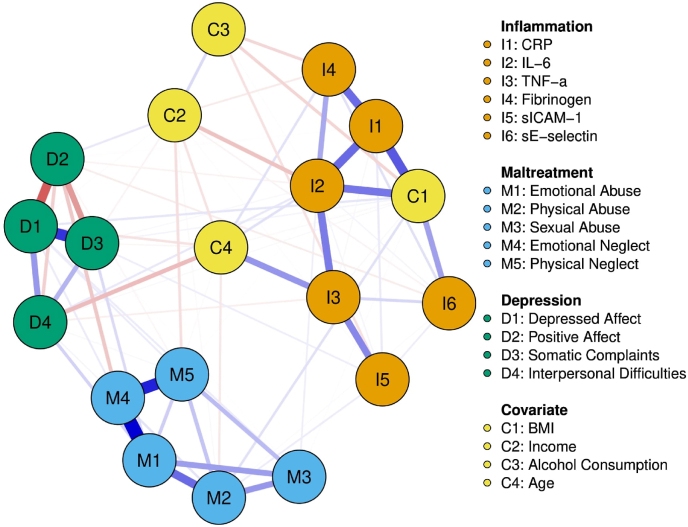
Network analysis of CM subtypes, MD symptom clusters, inflammatory molecules, and covariates for female participants Nodes represent variables and edges represent assocaitons. Edges in red denote a negative association where as edges in blue denote a positive association. (For interpretation of the references to colour in this figure legend, the reader is referred to the Web version of this article.)

Fig. 2 depicts the male model and Fig. 3 depicts the female model. Both models consisted of 19 nodes, with the female model having 84 non-zero edges and a mean weight of 0.023 and the male model having 68 non-zero edges and a mean weight of 0.022. Strongest node clustering was still noted between nodes of the same construct. Strong associations for males were observed between depressed affect and somatic complaints (0.366), emotional abuse and emotional neglect (0.341), emotional neglect and physical neglect (0.391), and CRP and fibrinogen (0.362). Strong associations for females were observed between emotional abuse and emotional neglect (0.438), emotional neglect and physical neglect (0.379), and depressed affect and somatic complaints (0.348). Across constructs, males had their strongest associations between IL-6 and physical neglect (0.022), and between IL-6 and somatic complaints (0.055). Females had their strongest associations across constructs between emotional abuse and TNF-α (0.061), IL-6 and interpersonal difficulties (0.040), and emotional neglect and positive affect (−0.094).

Reliability analyses indicated that node centrality strength had excellent stability for males CS(cor = 0.7) = 0.750 and females CS(cor = 0.7) = 0.750. Nodes with the highest strength centrality for males were somatic complaints (1.400), IL-6 (1.431), CRP (1.277), emotional abuse (1.205), depressed affect (1.137), and emotional neglect (1.053). However, emotional abuse (1.575), IL-6 (1.630), and depressed affect (1.103) were the strongest for females. Reliability analyses for node closeness were acceptable but should be interpreted with care for males (CS (cor = 0.7) = 0.361) and females (CS (cor = 0.7) = 0.439). High centrality closeness was observed among IL-6 (1.453) and age (1.774) for males, and among IL-6 (1.469), TNF-α (1.078), BMI (1.627), and age (1.152) for females. Reliability analysis for betweenness centrality was weak in both models and should not be interpreted as reliable (male = 0.125, female = 0.127). All network edge coefficients, plotted bootstrapped edges, and centrality coefficients can be reviewed in supplemental materials (Supplemental Figs. 3–5, Supplemental Tables 4–6).

### 3.5 Network comparison testing

The network comparison test between male and female models revealed significant model invariance (M = 0.149, p = 0.01), but not significant differences in global network strength (p = 0.26). Edge invariance testing revealed that there were 16 edges that differed: CRP and TNF-α (p = 0.05), CRP and fibrinogen (p < 0.01), fibrinogen and sE-selectin (p < 0.01), sICAM-1 and sE-selectin (p = 0.01), emotional abuse and sexual abuse (p = 0.01), physical abuse and sexual abuse (p = 0.01), sexual abuse and emotional neglect (p = 0.02), sE-selectin and somatic complaints (p = 0.05), IL-6 and interpersonal difficulties (p = 0.04), IL-6 and BMI (p < 0.01), depressed affect and BMI (p < 0.01), interpersonal difficulties and income (p = 0.001), IL-6 and age (p < 0.01), TNF-α and age (p = 0.01), sE-selectin and age (p = 0.01), and somatic complaints and age (p = 0.03). Notably, the associations between IL-6 and physical neglect, IL-6 and somatic complaints, TNF- α and emotional abuse, and emotional neglect and positive affect did not approach statistical significance, indicating no significant differences between the male and female networks on these specific edges.

### 3.6 Sensitivity analysis

To test the sensitivity of multivariate results to the inclusion of CRP levels greater than 10 μg/mL we retested the total sample mode, including individuals with CRP levels as high as 79.3 μg/mL. This shifted mean CRP levels to 2.99. The expanded network model was found to have 93 non-zero edges, with the majority of new edges being relatively small in magnitude, and the most prominent new edge being between income and interpersonal difficulties (−0.026). A full review of edge weights can be located in Supplemental Table 7.

## 4 Discussion

The present study is the first to our knowledge to incorporate the effects of CM in the complex relationship between inflammation and MD using a network model, as well as the first to compare these networks disaggregated by sex. While several studies have explored the relationships between these constructs using more directed methods, like regression and structural equation modeling, the use of network analysis allows for the analysis of high dimensional data and complex relationships that are inherent in these constructs.

Results of the present study support some of the findings of both Fried et al. (2020) and Moriarity et al. (2021). Fried et al. (2020) noted an association between IL-6 and ‘aches and pains,’ and ‘sleep too much,’ which aligns with an association between IL-6 and somatic complaints in the present study. Thus, our results continue to support the conceptual replication of Jokela et al. (2016) who identified that increased CRP is associated with specific depressive symptoms such as fatigue, sleep disturbance, anhedonia, and appetite disturbance (i.e. sickness behavior). Somatic complaints may then confer a risk for an episode of MD as supported by Moriarity et al.’s (2021) finding that inflammation is associated with connectivity between depressive symptoms. Results from the total network model support this line of inquiry showing a positive association between CRP and IL-6, and that IL-6 was associated with somatic complaints which is significantly associated with other depressive symptoms.

While previous network analyses examined how inflammation was related to MD, the present study innovated by including measures of CM, with several strong associations between maltreatment and depressive symptoms. Our finding that emotional abuse has strong strength centrality and is well connected to depressive symptom clusters across models supports the conclusions of a network analysis by Zhou et al. (2022) who found experiences of emotional abuse to be the most consequential factor for major depression. Additionally, the strong association between emotional abuse and other forms of CM such as physical abuse and emotional neglect support the recognition that CM associated with major depression is often complex and not characterized by a single type of CM (Teicher et al., 2022). These findings may also shed light on recent work exploring threat-deprivation dimensionality model in which experiences of threats, but not deprivation, were found to be associated with internalizing problems (such as MD) at age 17 (Miller et al., 2018).

It should also be noted that several small associations were noted between CM and inflammation markers, most notably sexual abuse and physical neglect were both associated with IL-6, and IL-6 was associated with somatic complaints. Thus, the total network model provides further support for the role of CM being associated with increased proinflammatory cytokines which are able to cross the BBB and affect the central nervous system to produce sickness behavior (Slavich and Irwin, 2014). Further, the total network model revealed IL-6 to be significantly associated with TNF- α, and a clustering of associations between TNF- α, sICAM-1, and sE-selectin. Importantly, TNF- α, sICAM-1, and sE-selectin have been implicated in potential hyperpermeability of the BBB, and the experience of psychosocial stress has been associated with modulation of the BBB (Cheng et al., 2018; Dion-Albert et al., 2022; Menard et al., 2017; Müller, 2019). Thus, while there is weak clustering between reported physical abuse, somatic complaints, sICAM-1, and sE-selectin, these associations may best be understood as the experience of psychosocial stress creating a hyperpermeability in the BBB that strengthens the association between IL-6 and somatic complaints.

Despite these findings and the important iterations on previous models, sex disaggregated network models highlighted several important distinctions, not detectable otherwise. First, a stronger association between IL-6 and interpersonal difficulties emerged for females, while the association between IL-6 and somatic complaints weakened. However, the association between IL-6 and somatic complaints strengthened marginally for males, and no association was noted for between IL-6 and interpersonal difficulties. Further, the association between TNF-α and IL-6 was still present across males and females, although the magnitude of association was stronger for females. These findings contrast with the findings of Chalmers et al. (2022) who identified an association between Il-6 and MD for a female network, but not a male network, further highlighting the need to measure differences in how MD is experienced. However, results here can also be viewed as complimentary to the work of Chalmers et al. (2022) in that IL-6 and interpersonal difficulties had a significantly stronger association for females, underscoring the need to study sex differences in MD and inflammation. These findings also align with Moieni et al.’s (2015) work identifying that females have a greater decreased mood and social disconnection when exposed to an inflammatory challenge, and that social disconnection was positively associated with TNF-α and IL-6 for females only. Additionally, Slavich et al. (2020) identified that interpersonal stress, but not non-interpersonal stress, was associated with greater inflammation reactivity as measured by TNF-α and interleukin-1β that helped explain greater depressive symptoms in a sample of adolescent females.

Notably, sex disaggregated models also provide support for studies evaluating the role of stress in endothelial markers and major depression, components of inflammation that have been understudied in psychiatric disorders (Müller, 2019). Analyses of the Maastritch study sample have found general endothelial dysfunction, as well as the main effects of sICAM-1 and sE-selectin, are associated with increased depressive symptoms (van Dooren et al., 2016; Geraets et al., 2020). Further some studies have found associations between endothelial markers and symptom clusters. A study comparing individuals with MD and high inflammation against individuals who have MD and low inflammation and healthy controls found higher concentrations of sICAM-1and IL-6 in the MD-high inflammation group. The MD-high inflammation group was also found to have lower BOLD signaling in areas of the brain related to reward anticipation (Burrows et al., 2021). Additionally, targeted BBB alterations at the prefrontal cortex of female mice have been associated with depressive phenotype, and similar BBB alterations have been found in mice exposed to a stress protocol (Dion-Albert et al., 2022). These alterations were similar to BBB morphology and were associated with increase sE-selectin in post-mortem women with a history of MD (Dion-Albert et al., 2022). The present study aligns with these findings in that several associations were noted between sICAM-1 and sE-selectin; however, associations were notably different between males and females. The present study found that males showed a greater association between sE-selectin and somatic complaints and interpersonal difficulties, while females had stronger associations between sE-selectin and positive affect and between sICAM-1 and somatic complaints. Although, it should also be considered that males had a stronger association between sE-selectin and sICAM-1 in the present study, and thus effects may be related to system level differences rather than individual molecules. Although, some evidence suggests vigorous exercise, which negatively correlates with sICAM-1, may relieve depressive symptoms for males only, suggesting potential sex differences in the role of endothelial markers (Cahuas et al., 2020; Many et al., 2013).

Last, it should also be considered that results for the present study may offer important contributions for furthering the treatment and prevention of MD. Across all models a connection between emotional abuse and somatic complaints and between emotional abuse and interpersonal difficulties, as well as a negative association between age and depressed affect and interpersonal difficulties was noted. While not all individuals who experience CM will go on to experience MD, the identification of emotional abuse by clinicians or child protective services, followed by the onset of interpersonal difficulties or somatic complaints may be a key warning sign for the development of a full depressive episode. Several randomized control trials have attempted preventative strategies for MD, and the development of treatment strategies aimed at preventing MD associated with CM through the targeting of interpersonal difficulties or somatic complaints may provide significant benefit given the often-recurrent nature of MD (de Jonge-Heesen et al., 2020; de Jonge-Heesen et al., 2016; Merry et al., 2011). Additionally, given MD is twice as likely to occur in females, the development of treatment methods specific to females is of high concern. Meta-analytic results from 56 randomized control trials identified that psychosocial interventions, particularly among those participating in cognitive behavioral therapy, were associated with lower levels of IL-6 and CRP (Shields et al., 2020). Additional meta-analytic results of 19 randomized control trials identified that psychosocial interventions most effectively reduced CRP in individuals experiencing psychological distress (O'Toole et al., 2018). Future studies aiming to improve the treatment of MD for females with a history CM that report interpersonal difficulties should evaluate the potential anti-depressive and anti-inflammatory effects of cognitive behavioral therapy.

### 4.1 Limitations

The present study makes significant strides in our understanding of MD; however, results should be tempered by several limitations. First, data are cross-sectional. It could be argued that CM affects inflammation which affects MD, although longitudinal data and improved study design are needed to draw this conclusion. Frequently, studies have failed longitudinal replication, and the role between inflammation and MD is hypothesized to be bidirectional (Beurel et al., 2020; Maxwell and Cole, 2007). Future studies should aim to utilize longitudinal approaches to psychometric network modeling to better understand how constructs influence each other over time and better highlight causal pathways. Second, estimation of network models via EBICglasso using Spearman's ρ allowed for estimating models with minimal data transformation, although this method does not allow for the use of categorical data. The unmeasured effects of smoking status and racial/ethnic identity, both of which may play a role in the measurement of depressive symptoms and inflammation, should be considered (Gonçalves et al., 2011; Mezuk et al., 2013). Future studies may aim to use a mixed-graphical-model approach while fitting data to a normal distribution using the non-paranormal package in order to build on the results presented here (Fried et al., 2020). Third, some evidence suggests poor overlap between prospective and retrospective histories of CM, with only half of reports being in agreement (Baldwin et al., 2019). Those who report experiencing CM are more likely to experience a mental health problem regardless of their subjective recall, whereas individuals who have an objective history of CM without subjective recall of CM were less likely to experience a mental health problem (Danese and Widom, 2020). Furthermore, the recall of CM as measured by the CTQ has been found to be stable over time and only marginally affected by current MD symptom levels, particularly as it pertains to the emotional abuse recall (Goltermann et al., 2021). Thus, broad generalization to individuals who have an objective history of CM should be made with care until subjective recall is confirmed, as individuals who are experiencing MD may have more prominent etiological mechanisms. Last, the present study utilizes the term “sex” to describe differences between individuals that identify as male vs female; however, this lacks precision. In terms of biological differences, variance in sex hormones that occur naturally over the lifespan such as during adolescence or at the start of menopause may be associated with differences in the expression of CRP (Slavich and Sacher, 2019). Further, the use of male-female sex is difficult to disentangle from the wide social influences associated with an individual's gender. Notably, females are more likely to experience negative life events than males and are more likely to appraise life events negatively during adolescence when discrepancies in the occurrence between male and female rates of MD first arise (Hyde et al., 2008). Although, it should also be considered that androgynous gender roles, as opposed to more masculine or more feminine, have been found to have a lower risk for MD as evidenced by meta-analytic results from 58 studies (Lin et al., 2021). Thus, psychological and sociological mechanisms appear to interact with biological mechanisms that require significant further work to fully elucidate.

## 5 Conclusion

The present study is the first to utilize a network analysis approach to examine how CM is associated with inflammation and MD and that these networks are different for males and females. Results confirm some of the well identified findings of the field such as the role of proinflammatory cytokines in depressive symptoms, as well as the strong associations between CM and MD. We also identified that several inflammatory markers were differently associated with depressive symptoms across males and females, indicating potential inflammation dimorphisms. Future studies at the intersection of CM and MD may benefit from evaluating if interventions affect the inflammation system in males and females differently, as well as potential moderating effects of endothelial markers.

## Financial Support

This research received no specific grant from any funding agency, commercial or not-for-profit sectors.

## Declaration of competing interest

None.

## Data Availability

Data is cited and publicly available via the MIDUS website or the ICPSR website
