# Supplementary material for: Sex differences in childhood maltreatment, inflammation, and adulthood depression: A network analysis
Source: Brain Behav Immun Health. 2023 Mar 1;29:100611. doi: 10.1016/j.bbih.2023.100611 (PMC10017358; doi:10.1016/j.bbih.2023.100611)
Supplement: Multimedia component 1 [file mmc1.docx]

**Supplemental Tables & Figures**

| *Supplemental Table 1: Laboratory methods for inflammatory markers* | | | | |
| --- | --- | --- | --- | --- |
|  | Sample Collected | Assay Method | Assay Range | Variability |
| *CRP^a^  *CRP^b^ | Citrated plasma  Serum | Immunoturbidometric  (BN II nephelometer; Dade Behring)  Immunoelectrochemiluminescent  (Meso Scale Diagnostics) | 0.164-800ug/mL  0.014-216/ug/mL | Inter: 1.08-4.3%  Intra: 2.3-4.4%  Inter: 4.72-5.16%  Intra: 2.2-4.1% |
| IL-6 | Serum | Immunoelectrochemiluminescent  (Meso Scale Diagnostics) | 1.58-488 pg/mL | Inter: 5-15%  Intra: 4.73% |
| TNF-α | Serum | Immunoelectrochemiluminescent  (Meso Scale Diagnostics) | 0.69-248 pg/mL | Inter: 7%  Intra: 3.19% |
| Fibrinogen | Citrated plasma | Immunoturbidometric  (BN II nephelometer; Dade Behring) | 2.8-4560 mg/dL | Inter: 4.13-6.64%  Intra: 2.7% |
| sICAM-1 | Serum | ELISA (R&D Systems) | 31-1000 ng/ML | Inter: 7.49-8.16%  Intra: 3.7-5.2% |
| sE-selectin | Serum | ELISA (R&D Systems) | 1.25-80 ng/mL | Inter: 7.1-11.15%  Intra: 5.2-6.6% |
| * Samples across both MIDUS 2 and MIDUS Refresher that fall below assay methods for CRP^a^ were reanalyzed using assay methods for CRP^b^. Beginning in 2016 all CRP assay methods utilized the methods for CRP^b^. | | | | |

| *Supplemental Figure 1:Bootstrapped reliability analysis for edge weights of the total network model*  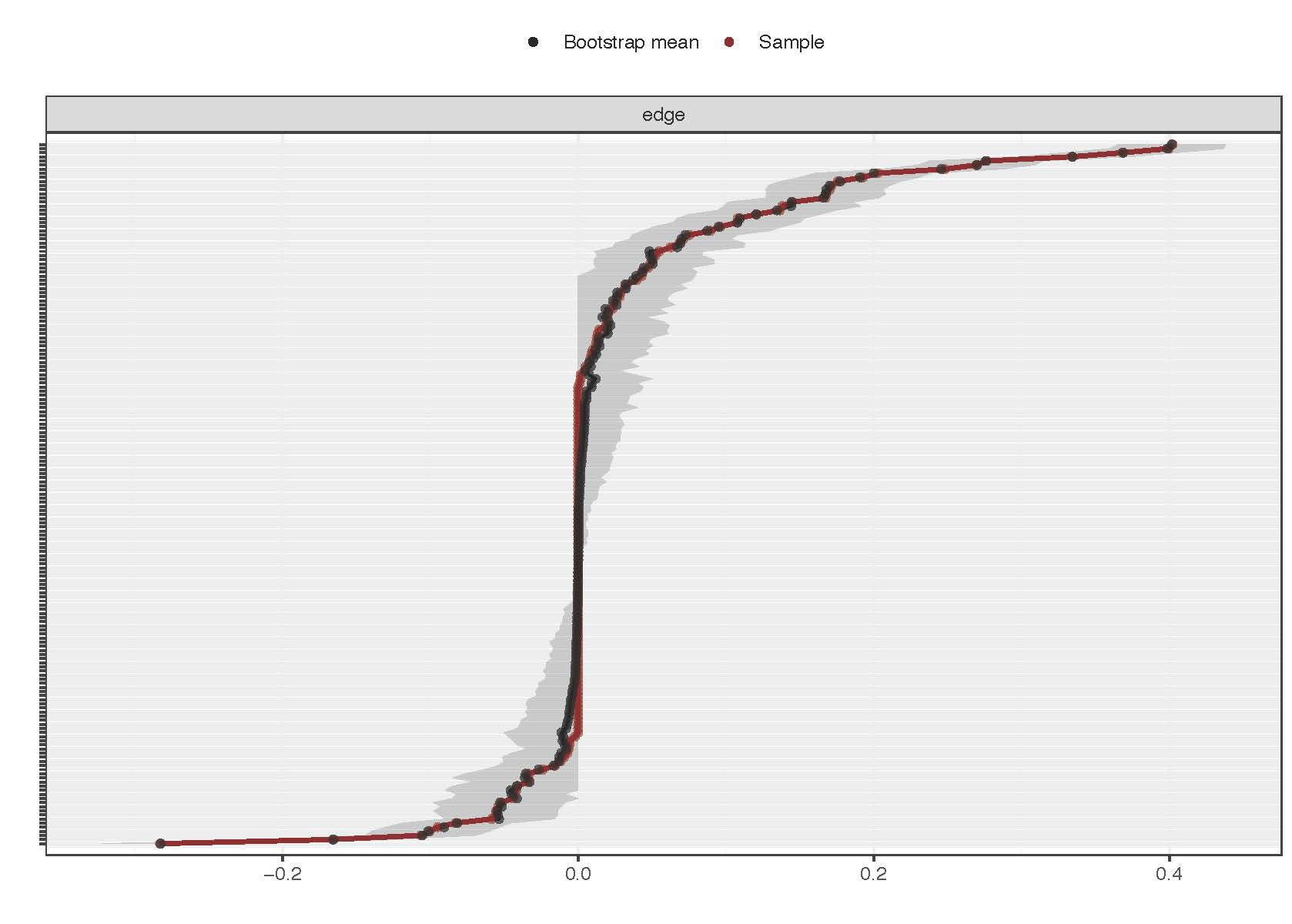  *Supplemental Figure 2: Bootstrapped reliability analysis for centrality measures of the total network model* |
| --- |
| 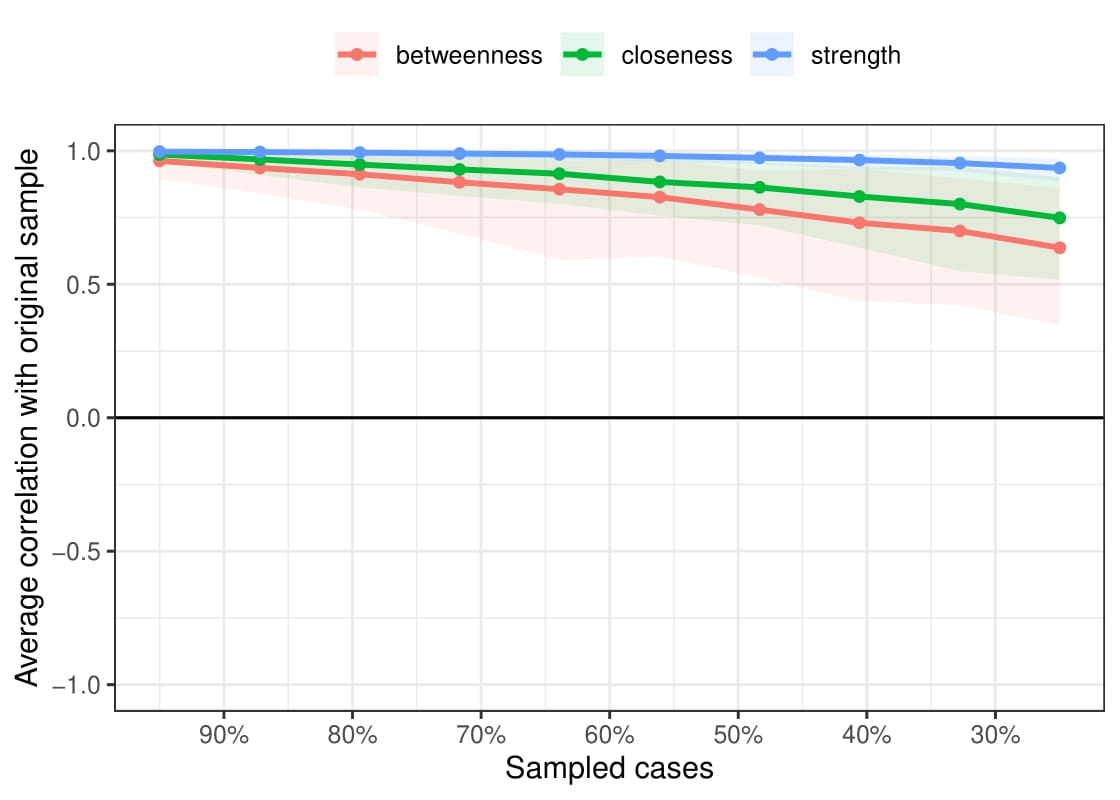 |

| *Supplemental table 2: Centrality measures for the total network model* | | | |  |
| --- | --- | --- | --- | --- |
|  | Strength | Closeness |  | |
| CRP | 1.104 | 0.926 |  | |
| IL-6 | 1.692 | 1.598 |  | |
| TNF-α | -0.345 | 0.468 |  | |
| Fibrinogen | -0.338 | -0.220 |  | |
| sICAM-1 | -1.026 | -1.122 |  | |
| sE-selectin | -1.046 | -0.984 |  | |
| Emotional abuse | 1.538 | 0.268 |  | |
| Physical abuse | -0.276 | -0.129 |  | |
| Sexual abuse | -1.159 | -1.587 |  | |
| Emotional neglect | 0.950 | -0.059 |  | |
| Physical neglect | 0.387 | -0.552 |  | |
| Depressed affect | 1.161 | 0.474 |  | |
| Positive affect | -0.202 | 0.208 |  | |
| Somatic complaints | 0.710 | -0.071 |  | |
| Interpersonal | -0.338 | 0.772 |  | |
| BMI | 0.183 | 1.056 |  | |
| Income | -1.085 | -0.130 |  | |
| Alcohol | -1.896 | -2.295 |  | |
| Age | 0.044 | 1.378 |  | |
| CRP: C-reactive protein; IL-6: interleukin-6; TNF-α: tumor necrosis factor-alpha; sICAM-1: soluble intercellular adhesion molecule-1; sE-selectin: soluble E-selectin; BMI: body mass index | | | |  |
|  | | | |  |

| *Supplemental Table 3: Edge coefficients for the total network model* | | | | | | | | | | | | | | | | | | | |
| --- | --- | --- | --- | --- | --- | --- | --- | --- | --- | --- | --- | --- | --- | --- | --- | --- | --- | --- | --- |
|  | I1 | I2 | I3 | I4 | I5 | I6 | M1 | M2 | M3 | M4 | M5 | D1 | D2 | D3 | D4 | C1 | C2 | C3 | C4 |
| I1 | - |  |  |  |  |  |  |  |  |  |  |  |  |  |  |  |  |  |  |
| I2 | 0.276 | - |  |  |  |  |  |  |  |  |  |  |  |  |  |  |  |  |  |
| I3 | 0.023 | 0.202 | - |  |  |  |  |  |  |  |  |  |  |  |  |  |  |  |  |
| I4 | 0.334 | 0.136 | - | - |  |  |  |  |  |  |  |  |  |  |  |  |  |  |  |
| I5 | 0.054 | 0.0009 | 0.192 | - | - |  |  |  |  |  |  |  |  |  |  |  |  |  |  |
| I6 | - | 0.042 | 0.068 | - | 0.096 | - |  |  |  |  |  |  |  |  |  |  |  |  |  |
| M1 | - | - | - | - | - | - | - |  |  |  |  |  |  |  |  |  |  |  |  |
| M2 | - | - | - | - | 0.019 | 0.014 | 0.269 | - |  |  |  |  |  |  |  |  |  |  |  |
| M3 | - | 0.012 | - | 0.009 | - | - | 0.167 | 0.120 | - |  |  |  |  |  |  |  |  |  |  |
| M4 | - | - | - | - | - | - | 0.399 | 0.050 | 0.011 | - |  |  |  |  |  |  |  |  |  |
| M5 | - | 0.019 | - | - | - | - | 0.048 | 0.107 | 0.089 | 0.402 | - |  |  |  |  |  |  |  |  |
| D1 | 0.004 | - | - | 0.082 | - | - | 0.043 | - | 0.025 | - | 0.028 | - |  |  |  |  |  |  |  |
| D2 | - | - | - | - | - | -0.005 | - | - | - | -0.106 | -0.023 | -0.282 | - |  |  |  |  |  |  |
| D3 | 0.007 | 0.047 | - | - | 0.033 | 0.004 | 0.071 | - | - | - | 0.012 | 0.368 | -0.166 | - |  |  |  |  |  |
| D4 | - | 0.008 | - | - | - | 0.020 | 0.075 | - | - | 0.001 | 0.028 | 0.171 | -0.055 | 0.167 | - |  |  |  |  |
| C1 | 0.248 | 0.175 | 0.018 | 0.031 | - | 0.170 | - | 0.052 | - | - | - | - | -0.006 | - | 0.023 | - |  |  |  |
| C2 | -0.007 | -0.095 | - | -0.015 | -0.044 | -0.002 | - | - | -0.011 | - | -0.058 | -0.009 | 0.039 | -0.033 | - | - | - |  |  |
| C3 | -0.005 | - | - | -0.054 | -0.041 | - | - | - | - | - | - | - | - | - | - | -0.055 | 0.108 | - |  |
| C4 | -0.033 | 0.138 | 0.144 | 0.062 | - | -0.051 | -0.043 | -0.033 | - | - | 0.013 | -0.082 | - | - | -0.100 | - | -0.041 | 0.001 | - |
| I1: C-reactive protein; I2: interlukin-6; I3: tumor necrotic factor-alpha; I4: Fibrinogen; I5: soluble intercellular adhesion molecule-1; I6: soluble E-selectin; M1: Emotional abuse; M2: Physical abuse; M3: Sexual abuse; M4: Emotional neglect; M5: Physical neglect; D1: Depressed affect; D2: Positive affect; D3: Somatic complaints; D4: Interpersonal; C1: BMI; C2: Income; C3: Alcohol; C4: Age  Edge coefficients representing the magnitude of association between two given nodes. Edge coefficients may be interpreted akin to partial correlations. Edge coefficients are calculated by multiplying an agency matrix by the weight matrix, thus any coefficients reported as - were noted as 0 in the agency matrix. | | | | | | | | | | | | | | | | | | | |

*Supplemental Figure 3: Bootstrapped reliability analysis for edge weights of the male network model*


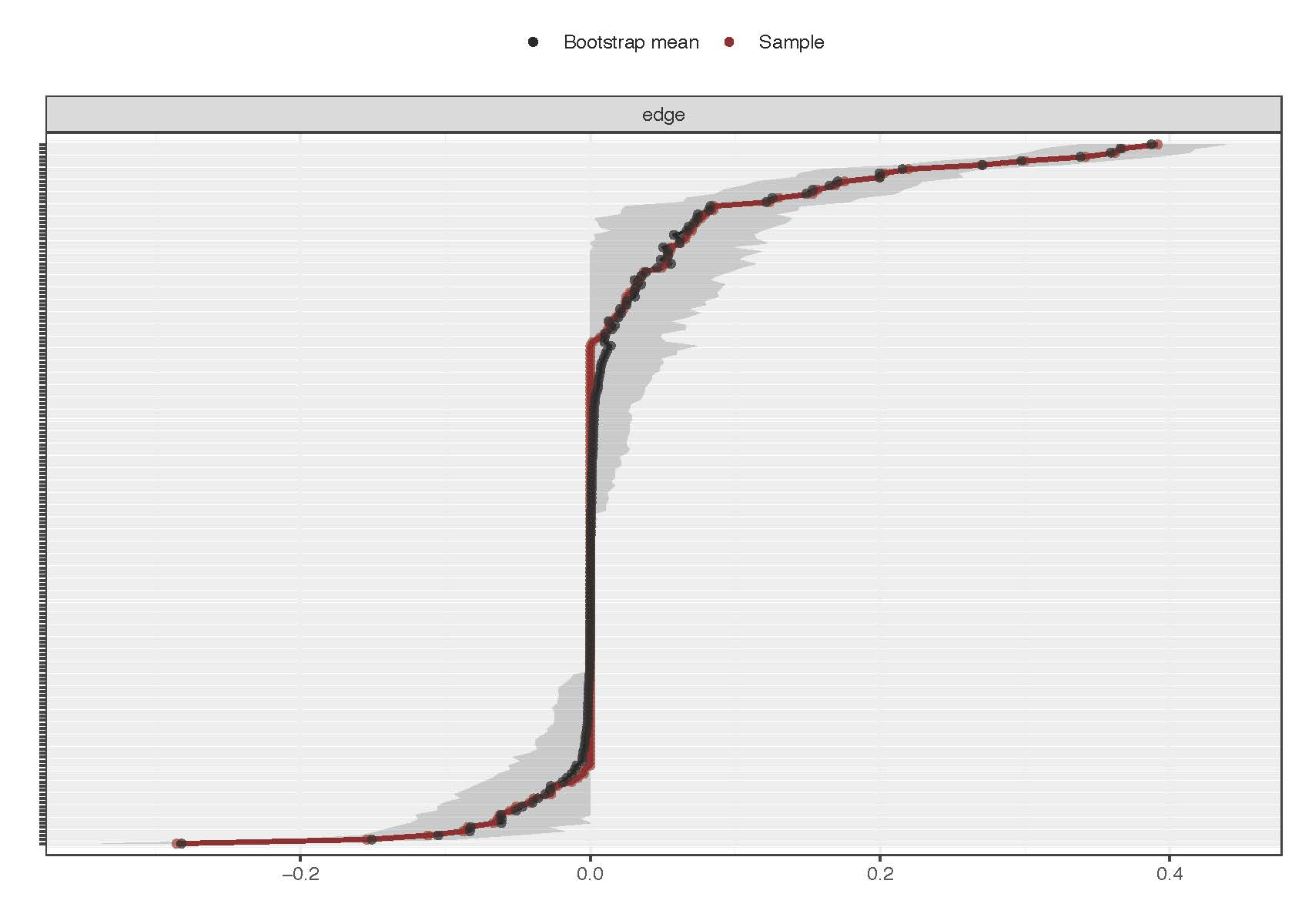


*Supplemental Figure 4: Bootstrapped reliability analysis for edge weights of female network models*


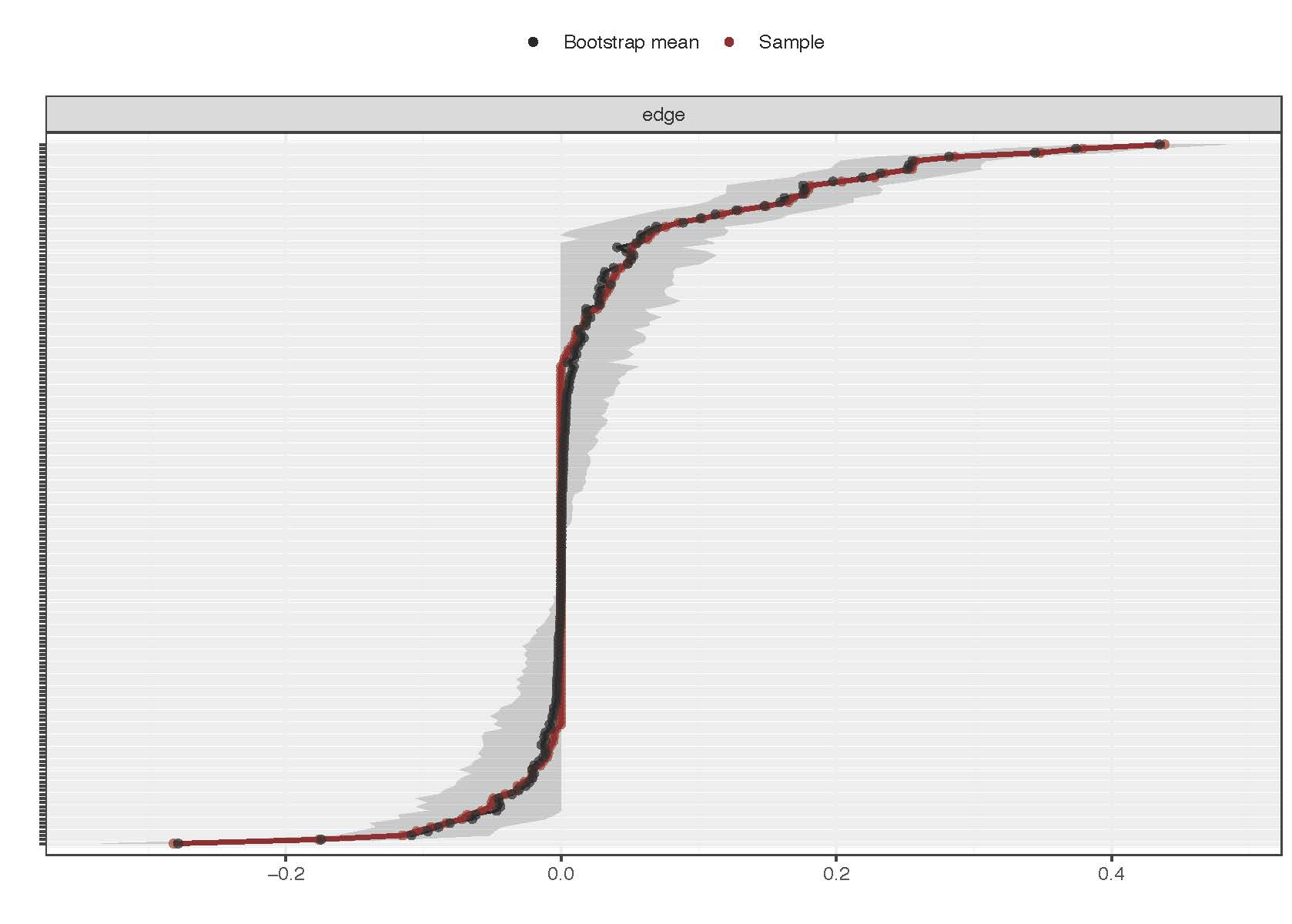


| *Supplemental Figure 5: Bootstrapped reliability analysis for centrality measures of the male (left) and female (right) network models* | |
| --- | --- |
| 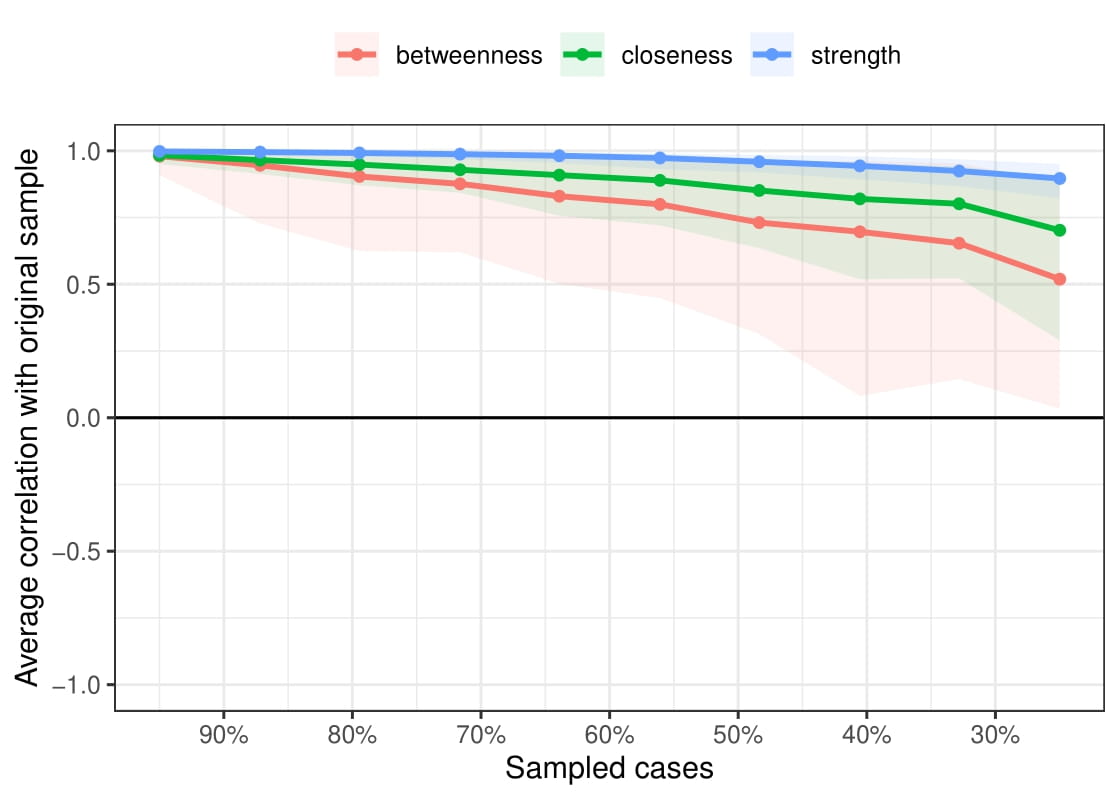 | 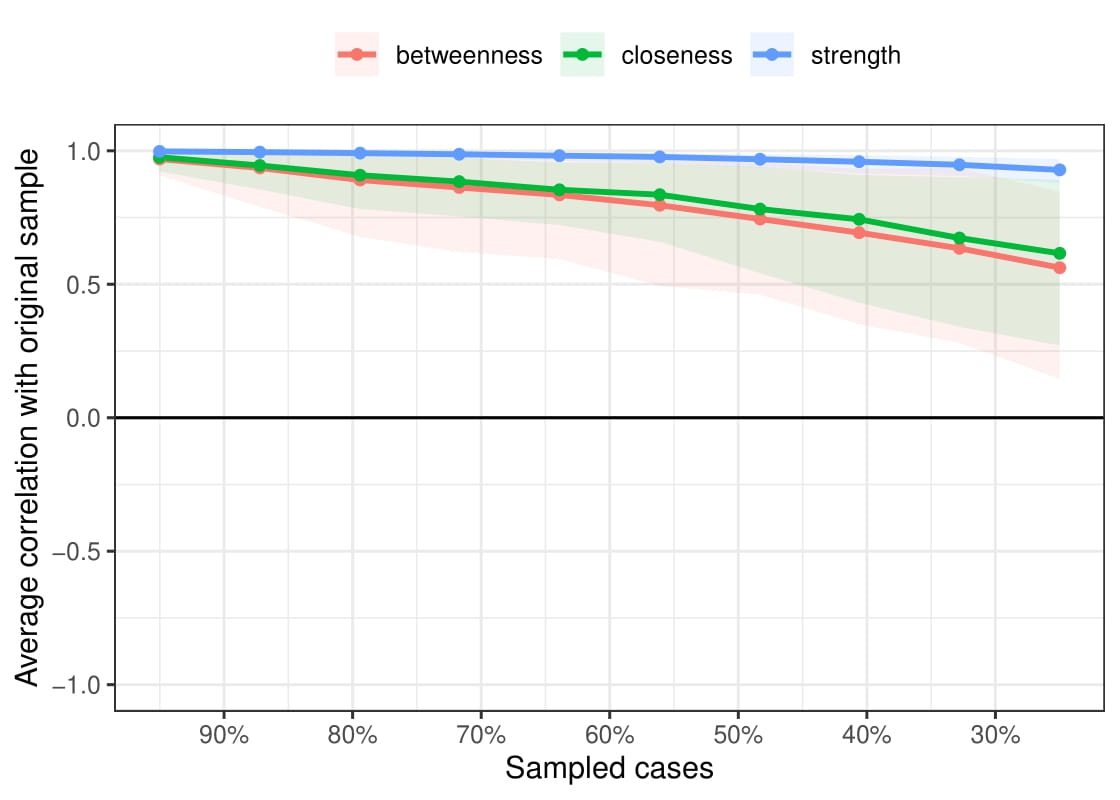 |

| *Supplemental table 4: Centrality measures for the sex disaggregated network models* | | | | | | |
| --- | --- | --- | --- | --- | --- | --- |
|  | Male Model | | | Female Model | | |
|  | Strength | Closeness |  | Strength | Closeness |  |
| CRP | 1.277 | 0.667 |  | 0.481 | 0.768 |  |
| IL-6 | 1.431 | 1.453 |  | 1.630 | 1.469 |  |
| TNF-α | -0.512 | -0.022 |  | 0.002 | 1.078 |  |
| Fibrinogen | -0.321 | -0.128 |  | -0.264 | -0.491 |  |
| sICAM-1 | -0.737 | -0.808 |  | -1.279 | -0.725 |  |
| sE-selectin | -0.438 | -0.249 |  | -1.327 | -0.911 |  |
| Emotional abuse | 1.205 | 0.485 |  | 1.575 | 0.218 |  |
| Physical abuse | -0.484 | -0.353 |  | -0.017 | 0.046 |  |
| Sexual abuse | -1.467 | -2.102 |  | -0.767 | -1.159 |  |
| Emotional neglect | 1.053 | 0.185 |  | 0.983 | -0.113 |  |
| Physical neglect | 0.377 | -0.188 |  | 0.293 | -0.575 |  |
| Depressed affect | 1.137 | 0.879 |  | 1.103 | 0.075 |  |
| Positive affect | -0.153 | 0.436 |  | 0.067 | -0.289 |  |
| Somatic complaints | 1.400 | 0.726 |  | 0.289 | -0.621 |  |
| Interpersonal | -0.361 | 0.081 |  | -0.088 | 0.863 |  |
| BMI | -0.504 | -0.215 |  | 0.988 | 1.627 |  |
| Income | -1.015 | -0.383 |  | -1.210 | -0.055 |  |
| Alcohol | -1.850 | -2.239 |  | -1.832 | -2.355 |  |
| Age | -0.036 | 1.774 |  | -0.630 | 1.152 |  |
| CRP: C-reactive protein; IL-6: interleukin-6; TNF-α: tumor necrosis factor-alpha; sICAM-1: soluble intercellular adhesion molecule-1; sE-selectin: soluble E-selectin; BMI: body mass index | | | | | | |
|  | | | | | | |

| *Supplemental Table 5: Edge coefficients for the male network model* | | | | | | | | | | | | | | | | | | | | |
| --- | --- | --- | --- | --- | --- | --- | --- | --- | --- | --- | --- | --- | --- | --- | --- | --- | --- | --- | --- | --- |
|  | I1 | I2 | I3 | I4 | I5 | I6 | M1 | M2 | M3 | M4 | M5 | D1 | D2 | D3 | D4 | C1 | C2 | C3 | C4 |  |
| I1 | - |  |  |  |  |  |  |  |  |  |  |  |  |  |  |  |  |  |  |  |
| I2 | 0.269 | - |  |  |  |  |  |  |  |  |  |  |  |  |  |  |  |  |  |  |
| I3 | 0.065 | 0.175 | - |  |  |  |  |  |  |  |  |  |  |  |  |  |  |  |  |  |
| I4 | 0.362 | 0.123 | - | - |  |  |  |  |  |  |  |  |  |  |  |  |  |  |  |  |
| I5 | 0.052 | - | 0.153 | - | - |  |  |  |  |  |  |  |  |  |  |  |  |  |  |  |
| I6 | 0.024 | 0.049 | 0.030 | - | 0.156 | - |  |  |  |  |  |  |  |  |  |  |  |  |  |  |
| M1 | - | - | - | - | - | - | - |  |  |  |  |  |  |  |  |  |  |  |  |  |
| M2 | - | - | - | - | - | 0.001 | 0.299 | - |  |  |  |  |  |  |  |  |  |  |  |  |
| M3 | - | - | - | - | - | - | 0.070 | 0.073 | - |  |  |  |  |  |  |  |  |  |  |  |
| M4 | - | - | - | - | - | - | 0.341 | 0.036 | 0.053 | - |  |  |  |  |  |  |  |  |  |  |
| M5 | - | 0.022 | - | - | - | - | 0.031 | 0.084 | 0.054 | 0.391 | - |  |  |  |  |  |  |  |  |  |
| D1 | - | - | - | - | - | - | 0.035 | - | - | 0.006 | 0.017 | - |  |  |  |  |  |  |  |  |
| D2 | - | - | - | - | -0.004 | - | - | - | - | -0.112 | -0.022 | -0.285 | - |  |  |  |  |  |  |  |
| D3 | 0.009 | 0.055 | - | - | 0.013 | 0.033 | 0.069 | - | - | - | 0.066 | 0.366 | -0.154 | - |  |  |  |  |  |  |
| D4 | - | - | - | - | - | 0.020 | 0.079 | - | - | - | 0.013 | 0.169 | -0.008 | 0.202 | - |  |  |  |  |  |
| C1 | 0.219 | 0.085 | 0.003 | 0.027 | - | 0.129 | - | 0.012 | - | - | - | - | - | - | - | - |  |  |  |  |
| C2 | - | -0.063 | -0.027 | - | -0.066 | - | - | - | - | - | -0.051 | - | 0.024 | -0.039 | - | - | - |  |  |  |
| C3 | - | - | - | - | -0.004 | - | - | - | - | - | - | - | - | 0.024 | - | -0.041 | 0.076 | - |  |  |
| C4 | - | 0.199 | 0.061 | 0.052 | - | -0.087 | -0.055 | -0.012 | - | - | - | -0.084 | - | - | -0.062 | - | -0.027 | - | - |  |
| I1: C-reactive protein; I2: interlukin-6; I3: tumor necrotic factor-alpha; I4: Fibrinogen; I5: soluble intercellular adhesion molecule-1; I6: soluble E-selectin; M1: Emotional abuse; M2: Physical abuse; M3: Sexual abuse; M4: Emotional neglect; M5: Physical neglect; D1: Depressed affect; D2: Positive affect; D3: Somatic complaints; D4: Interpersonal; C1: BMI; C2: Income; C3: Alcohol; C4: Age  Edge coefficients representing the magnitude of association between two given nodes. Edge coefficients may be interpreted akin to partial correlations. Edge coefficients are calculated by multiplying an agency matrix by the weight matrix, thus any coefficients reported as - were noted as 0 in the agency matrix. | | | | | | | | | | | | | | | | | | | | |

| *Supplemental Table 6: Edge coefficients for the female network model* | | | | | | | | | | | | | | | | | | | | |
| --- | --- | --- | --- | --- | --- | --- | --- | --- | --- | --- | --- | --- | --- | --- | --- | --- | --- | --- | --- | --- |
|  | I1 | I2 | I3 | I4 | I5 | I6 | M1 | M2 | M3 | M4 | M5 | D1 | D2 | D3 | D4 | C1 | C2 | C3 | C4 |  |
| I1 | - |  |  |  |  |  |  |  |  |  |  |  |  |  |  |  |  |  |  |  |
| I2 | 0.257 | - |  |  |  |  |  |  |  |  |  |  |  |  |  |  |  |  |  |  |
| I3 | 0.010 | 0.227 | - |  |  |  |  |  |  |  |  |  |  |  |  |  |  |  |  |  |
| I4 | 0.254 | 0.149 | - | - |  |  |  |  |  |  |  |  |  |  |  |  |  |  |  |  |
| I5 | 0.036 | 0.016 | - | - | - |  |  |  |  |  |  |  |  |  |  |  |  |  |  |  |
| I6 | - | 0.017 | 0.204 | 0.051 | 0.029 | - |  |  |  |  |  |  |  |  |  |  |  |  |  |  |
| M1 | - | - | 0.068 | - | - | - | - |  |  |  |  |  |  |  |  |  |  |  |  |  |
| M2 | - | - | - | - | 0.030 | 0.005 | 0.255 | - |  |  |  |  |  |  |  |  |  |  |  |  |
| M3 | - | 0.026 | - | - | - | - | 0.168 | 0.179 | - |  |  |  |  |  |  |  |  |  |  |  |
| M4 | - | - | - | - | - | - | 0.438 | 0.048 | - | - |  |  |  |  |  |  |  |  |  |  |
| M5 | - | - | - | - | - | - | 0.085 | 0.102 | 0.116 | 0.379 | - |  |  |  |  |  |  |  |  |  |
| D1 | - | - | - | - | - | - | 0.035 | - | 0.034 | - | 0.033 | - |  |  |  |  |  |  |  |  |
| D2 | -0.003 | - | - | - | - | -0.031 | - | - | - | -0.094 | -0.015 | -0.281 | - |  |  |  |  |  |  |  |
| D3 | - | 0.012 | - | - | 0.028 | - | 0.062 | - | 0.002 | - | - | 0.348 | -0.175 | - |  |  |  |  |  |  |
| D4 | - | 0.040 | -0.026 | - | - | - | 0.075 | - | - | 0.017 | 0.011 | 0.177 | -0.083 | 0.129 | - |  |  |  |  |  |
| C1 | 0.285 | 0.234 | 0.009 | 0.055 | - | 0.165 | - | 0.051 | 0.004 | - | - | 0.043 | -0.021 | 0.019 | 0.007 | - |  |  |  |  |
| C2 | - | -0.105 | - | -0.031 | -0.005 | -0.020 | - | -0.009 | - | - | -0.057 | -0.005 | 0.038 | -0.010 | -0.021 | - | - |  |  |  |
| C3 | - | -0.005 | - | -0.068 | -0.051 | - | - | - | - | - | - | - | - | - | - | -0.071 | 0.064 | - |  |  |
| C4 | - | 0.050 | 0.181 | 0.050 | - | - | -0.007 | -0.040 | - | - | 0.00005 | -0.050 | - | -0.013 | -0.115 | -0.009 | -0.049 | - | - |  |
| I1: C-reactive protein; I2: interlukin-6; I3: tumor necrotic factor-alpha; I4: Fibrinogen; I5: soluble intercellular adhesion molecule-1; I6: soluble E-selectin; M1: Emotional abuse; M2: Physical abuse; M3: Sexual abuse; M4: Emotional neglect; M5: Physical neglect; D1: Depressed affect; D2: Positive affect; D3: Somatic complaints; D4: Interpersonal; C1: BMI; C2: Income; C3: Alcohol; C4: Age  Edge coefficients representing the magnitude of association between two given nodes. Edge coefficients may be interpreted akin to partial correlations. Edge coefficients are calculated by multiplying an agency matrix by the weight matrix, thus any coefficients reported as - were noted as 0 in the agency matrix. | | | | | | | | | | | | | | | | | | | | |

| *Supplemental Table 7: Edge coefficients for the network model using the total sample, including CRP levels greater than 10* | | | | | | | | | | | | | | | | | | | | |
| --- | --- | --- | --- | --- | --- | --- | --- | --- | --- | --- | --- | --- | --- | --- | --- | --- | --- | --- | --- | --- |
|  | I1 | I2 | I3 | I4 | I5 | I6 | M1 | M2 | M3 | M4 | M5 | D1 | D2 | D3 | D4 | C1 | C2 | C3 | C4 |  |
| I1 | - |  |  |  |  |  |  |  |  |  |  |  |  |  |  |  |  |  |  |  |
| I2 | 0.297 | - |  |  |  |  |  |  |  |  |  |  |  |  |  |  |  |  |  |  |
| I3 | 0.018 | 0.202 | - |  |  |  |  |  |  |  |  |  |  |  |  |  |  |  |  |  |
| I4 | 0.365 | 0.140 | - | - |  |  |  |  |  |  |  |  |  |  |  |  |  |  |  |  |
| I5 | 0.057 | 0.003 | 0.191 | - | - |  |  |  |  |  |  |  |  |  |  |  |  |  |  |  |
| I6 | 0.001 | 0.050 | 0.073 | 0.001 | 0.091 | - |  |  |  |  |  |  |  |  |  |  |  |  |  |  |
| M1 | - | - | - | - | - | - | - |  |  |  |  |  |  |  |  |  |  |  |  |  |
| M2 | - | - | - | - | 0.012 | 0.021 | 0.278 | - |  |  |  |  |  |  |  |  |  |  |  |  |
| M3 | - | 0.014 | - | 0.025 | - | - | 0.173 | 0.127 | - |  |  |  |  |  |  |  |  |  |  |  |
| M4 | - | - | - | - | - | - | 0.403 | 0.043 | 0.014 | - |  |  |  |  |  |  |  |  |  |  |
| M5 | 0.006 | 0.021 | - | - | - | - | 0.050 | 0.113 | 0.074 | 0.406 | - |  |  |  |  |  |  |  |  |  |
| D1 | 0.0009 | - | - | 0.011 | - | - | 0.038 | - | 0.035 | - | 0.003 | - |  |  |  |  |  |  |  |  |
| D2 | - | - | - | - | - | -0.002 | - | - | - | -0.109 | -0.023 | -0.285 | - |  |  |  |  |  |  |  |
| D3 | 0.008 | 0.050 | - | - | 0.039 | 0.007 | 0.075 | - | - | - | 0.003 | 0.368 | -0.161 | - |  |  |  |  |  |  |
| D4 | - | 0.007 | -0.026 | - | - | 0.018 | 0.070 | - | - | 0.006 | 0.026 | 0.172 | -0.050 | 0.173 | - |  |  |  |  |  |
| C1 | 0.260 | 0.162 | 0.021 | 0.035 | - | 0.174 | - | 0.049 | - | - | - | - | -0.008 | - | 0.027 | - |  |  |  |  |
| C2 | -0.011 | -0.089 | -0.001 | -0.014 | -0.044 | -0.007 | - | -0.004 | -0.027 | - | -0.054 | -0.010 | 0.049 | -0.029 | - | - | - |  |  |  |
| C3 | -0.002 | - | - | -0.053 | -0.044 | - | - | - | - | - | - | - | - | - | - | -0.065 | 0.114 | - |  |  |
| C4 | -0.043 | 0.136 | 0.149 | 0.059 | - | -0.062 | -0.051 | -0.041 | - | - | 0.025 | -0.078 | 0.002 | - | -0.100 | -0.007 | -0.043 | 0.011 | - |  |
| I1: C-reactive protein; I2: interlukin-6; I3: tumor necrotic factor-alpha; I4: Fibrinogen; I5: soluble intercellular adhesion molecule-1; I6: soluble E-selectin; M1: Emotional abuse; M2: Physical abuse; M3: Sexual abuse; M4: Emotional neglect; M5: Physical neglect; D1: Depressed affect; D2: Positive affect; D3: Somatic complaints; D4: Interpersonal; C1: BMI; C2: Income; C3: Alcohol; C4: Age  Edge coefficients representing the magnitude of association between two given nodes. Edge coefficients may be interpreted akin to partial correlations. Edge coefficients are calculated by multiplying an agency matrix by the weight matrix, thus any coefficients reported as - were noted as 0 in the agency matrix. | | | | | | | | | | | | | | | | | | | | |
